# How aggressive interactions with biomimetic agents optimize reproductive performances in mass-reared males of the Mediterranean fruit fly

**DOI:** 10.1007/s00422-023-00965-w

**Published:** 2023-05-31

**Authors:** Donato Romano, Giovanni Benelli, Cesare Stefanini

**Affiliations:** 1 The BioRobotics Institute, Sant’Anna School of Advanced Studies, Viale Rinaldo Piaggio 34, 56025 Pontedera, Pisa, Italy; 2 Department of Excellence in Robotics and AI, Sant’Anna School of Advanced Studies, 56127 Pisa, Italy; 3 Department of Agriculture, Food and Environment, University of Pisa, Via del Borghetto 80, 56124 Pisa, Italy

**Keywords:** Animal–robot interaction, Bionics, Ethorobotics, Mass-rearing, Mediterranean fruit fly, Reproductive behaviour

## Abstract

Mass-rearing procedures of insect species, often used in biological control and Sterile Insect Technique, can reduce the insects competitiveness in foraging, dispersal, and mating. The evocation of certain behaviours responsible to induce specific neuroendocrine products may restore or improve the competitiveness of mass-reared individuals. Herein, we used a mass-reared strain of *Ceratitis capitata* as model organism. *C. capitata* is a polyphagous pest exhibiting territorial displays that are closely related to its reproductive performance. We tested if the behaviour of *C. capitata* males could be altered by hybrid aggressive interactions with a conspecific-mimicking robotic fly, leading to more competitive individuals in subsequent mating events. Aggressive interactions with the robotic fly had a notable effect on subsequent courtship and mating sequences of males that performed longer courtship displays compared to naïve individuals. Furthermore, previous interactions with the robotic fly produced a higher mating success of males. Reproductive performances of *C. capitata* males may be improved by specific octopaminergic neurones activated during previous aggressive interactions with the robotic fly. This study adds fundamental knowledge on the potential role of specific neuro-behavioural processes in the ecology of tephritid species and paves the way to innovative biotechnological control methods based on robotics and bionics.

**Supplementary Information:**

The online version contains supplementary material available at 10.1007/s00422-023-00965-w.

## Introduction

The growing attention for health and sustainability is launching new challenges to guarantee food security and environmental conservation (van der Goot et al. 2016; Garcia-Herrero et al 2018). The increased demand for more efficient and sustainable food processes, and the increased limitation of the use of chemicals require alternative methods for managing pests organisms (Radcliffe et al. 2009; Sørensen et al. 2012).

In recent times, the development of sustainable pest control strategies has become a major issue to reduce the impact of species of medical and/or economic importance. Sustainability of pest management programs can be pursued by several approaches, including biological control and Sterile Insect Technique (SIT), that contribute to reduce the inputs coming from non-renewable energy sources, as well as to minimize the adverse consequences to the ecosystem (Quimby et al. 2002; Hajek et al. 2018; Anguelov et al. 2020; Vreysen et al. 2006). Biological control relies on the use of natural enemies to reduce the population of a species considered as a pest (Eilenberg 2001; Cock et al. 2010). With particular reference to the augmentative biological control, natural enemies are mass-reared to be released in large numbers for breaking down the pest population (van Lenteren et al. 2018). SIT is a species-specific control method based on the mass-rearing, sterilization and release of large numbers of male insects (Alphey 2002) which when mating with native females produces a decrease in their reproductive potential. The massive release of these males over a sufficient period of time may lead to the local eradication of the pest population.

Thus, mass-rearing is a crucial component of both these pest management strategies. However, rearing conditions (and also sterilization procedures for SIT) often affect insect performance in terms of competitiveness, resulting in animals with less effective foraging, dispersal, and mating behaviours (Sørensen et al. 2012; Reger et al. 2021). To mitigate mass-reared insect poor performance, different strategies have been explored, mainly relying on thermal biology, phenotypic plasticity, and artificial selection (Sørensen et al. 2012).

Learning and experience as an adaptive response to field conditions can have a crucial role to increase quality and performance of mass-reared insects, changing paradigms for animal management. Indeed, more and more studies report that insects exhibit sophisticated behavioural repertoires, in spite of their miniature nervous systems (Sarin and Dukas 2009; Giurfa 2013; Perry 2017). Insect neural circuits allow them to learn and memorize various stimuli, exploiting this information in the form of experience in subsequent contexts in the short- and long-term range (Keene and Waddell 2007; Guerrieri and d’Ettorre 2010).

In this study, we propose a biorobotic-based approach for altering the behaviour of mass-reared insects via the interaction with biomimetic robotic agents, leading to experienced individuals with more competitive behaviours. Creating biohybrid colonies of animals and robots interacting each other represents an emergent context of bionics encompassing animal behavioural ecology and robotics (Romano et al. 2019). This relatively novel field of science and technology provides advanced engineered systems for studying assumptions on cognitive and ecological mechanisms in animals that can be generalized to humans, as well as to control intraspecific and interspecific interactions for applied purposes (Polverino et al. 2019; Jolles et al. 2020; Romano and Stefanini 2021; Worm et al. 2021). A growing number of studies are using biomimetic robots to interact with many animal species, ranging from invertebrates to vertebrates. Just to cite some examples, robotic agents have been used to interact with mammals (Kubinyi et al. 2004; Takanishi et al. 1998; Shi et al. 2015; Gianelli et al. 2018), birds (Patricelli et al. 2006; Butler and Fernández-Juricic 2014; Gribovskiy et al. 2018), reptiles (Brian Smith and Martins 2006; Partan et al. 2011), amphibians (Taylor et al. 2008), fish (Landgraf et al. 2016; Bierbach et al. 2018; Bonnet et al. 2018; Romano and Stefanini 2022; Polverino et al. 2022), insects (Halloy et al. 2007; Landgraf et al. 2011; Kawabata et al. 2014; Romano et al. 2021; Ilgun and Schmickl 2022), crustaceans (Rashid et al. 2012; Kawai and Gunji 2022; Romano et al. 2022), and arachnids (Benelli et al. 2018). Ethorobotics studies have explored several behaviour processes in animals, such as courtship behaviour (Romano et al. 2020), social affiliation (Langraf et al. 2016; Bonnet et al. 2018; Bierbach et al. 2020), social learning (Romano et al. 2021), agonistic interaction (Romano et al. 2017a), predator–prey interaction (Polverino et al. 2019), among others.

Herein, a mass-reared strain of the Mediterranean fruit fly (medfly), *Ceratitis capitata* Wiedemann (Diptera: Tephritidae) was used as elective model to investigate the effect of aggressive interaction of males with a conspecific-like artificial agent (hereafter robotic fly), on the subsequent mating success with females. The medfly is a polyphagous pest of major economic importance, attacking over 200 fruit species worldwide (Rasolofoarivao et al. 2021). This species is often mass-reared to release sterile males in SIT programs (Nikolouli et al. 2020), or to provide hosts for endoparasitoid species that are important for biological control (Benelli et al. 2013). So, the reproductive performance of mass-reared strain of this Tephritidae is relevant to both biological control and SIT techniques. *C. capitata* exhibits a highly ritualized aggressive display that is closely related to this species reproductive behaviour (Briceño et al. 1999). Males form leks, fight for territories used for courtship (e.g. on host and non-host plants), and attract females by releasing long-range pheromones (Papadopoulos et al. 2009; Benelli et al. 2014a, b, 2015). Generally, changes in agonistic and courtship behaviours are governed by changes within the central nervous system and depend on neuro-hormonal mechanisms. Territorial and reproductive behaviours may be linked thanks to the activation of specific neural circuits that modulate the level of neuroendocrine products following fighting interactions and physical exertion (Adamo et al. 1995). Females choose and copulate with those males that by performing courtship behaviour express their good quality (Whittier et al. 1994; Benelli and Romano 2018).

We used robotic flies to induce territorial behaviour in *C. capitata* males, and subsequently, we tested their courtship performance and mating success. We assessed whether male reproductive behaviour was affected by the previous animal–robot aggressive interaction experience, outperforming naïve males. We predicted that this biohybrid agonistic interaction may cause the activation of dedicated neuromodulators crucially affecting insect motivation and learning performances. Our bioengineered approach outlines the possibility to increase mating competitiveness of males, subject to mass-rearing procedures, by manipulating territorial skills via biomimetic robots during the pre-release phase.

## Materials and methods

### Ethical note

The present research adheres to the guidelines for the treatment of animals in behavioural research and teaching (ASAB/ABS 2014), the Italian laws (D.M. 116,192), and the regulations of the European Union (European Commission 2007). All the experiments consisted in behavioural observations. For tests involving *C. capitata,* no particular permits were needed by the Italian government.

### *Ceratitis capitata* mass-rearing and general observations

The *C. capitata* strain used in this study was mass-reared at the University of Pisa since 1994, staring from about 4000 wild medflies collected in fruit orchards (Sicily, Italy). The strain was constantly renewed adding wild flies in 1997, 2003, 2007, 2012, and 2016 (about 2000 flies per renewal, sex ratio 1:1). Cylindrical PVC cages were used as rearing production units. Each cage contained about 2000 flies (sex ratio 1:1). Adults diet consisted in a dry mixture of yeast extract and sucrose at a ratio of 1:10 (w:w). A cotton wick provided separately water. Eggs collection occurred every 2 days. Plastic bowls (50 × 15 cm and 2 cm high), each containing 500 g of artificial larval food medium, were used to place eggs. Before adult emergence, pupae were maintained under controlled conditions (21 ± 1 °C, 55 ± 5% relative humidity, 16:8 h L:D).

Experiments were conducted at the BioRobotics Institute of Scuola Superiore Sant’Anna (Pisa), at 21 ± 1 °C, and 55 ± 5% relative humidity. Fluorescent daylight tubes (16:8 h light:dark, lights on at 0600) were used for illumination. A LI-1800 spectroradiometer (LI-COR Inc., Lincoln, NE, U.S.A.), equipped with a remote cosine receptor, measured light intensity around the test arena that was ca. 1000 lx, estimated over the 300–1100-nm waveband. Diffuse laboratory lighting was used to limit reflection and directional light cues causing phototaxis.

Emerged *C. capitata* adults were sexed and individually placed in clean Plexiglas cups (diameter: 40 mm; length: 7 mm). *C. capitata* is a sexual dimorphic species. In particular, adult males present sexually dimorphic fronto-orbital bristles including a spatula-shaped terminal end. Adult females have a well visible ovipositor at the distal part of the abdomen (Diesner et al. 2022). Food and water were supplied similarly to the mass-rearing phase. In all experiments, virgin mature medflies (age 12–18 days old) were used, considering that gonad maturation is completed at 4–6 days from emergence in *C. capitata* (Shelly 2000). For each replicate, new medflies of the same age were used.

### Robotic fly and experimental apparatus

The robotic fly was inspired by the morphology, size, and colours of *C. capitata* adults, and included the head (with two compound eyes and two antennae), the thorax (whit three pairs of legs and one pair of wings), and the abdomen. The distance between the distal end of the head and the abdomen was 5 mm, and the distance between the tips of the two wings was 9 mm.

**Fig. 1:**
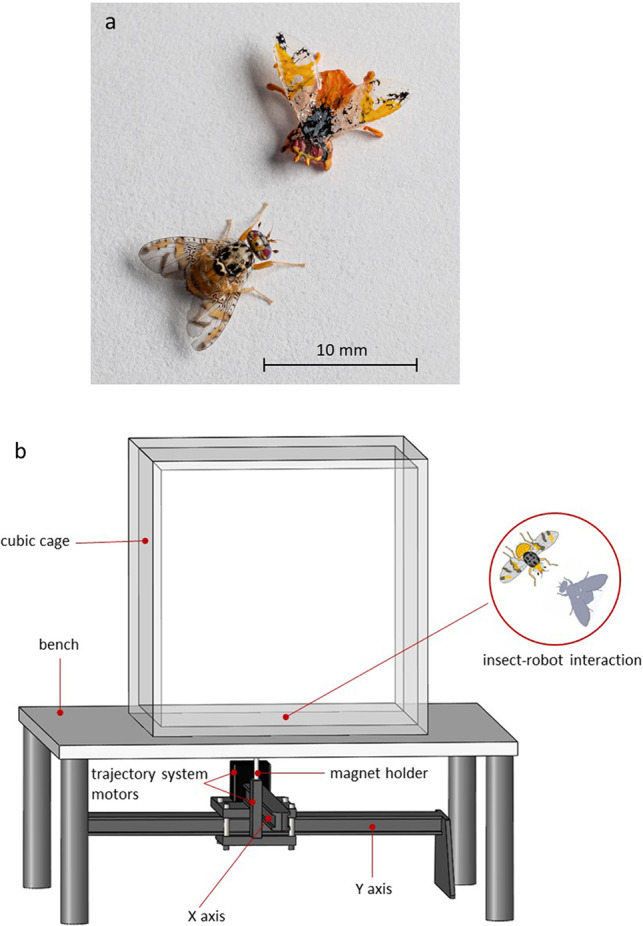
Robotic fly and a *Ceratitis capitata* adult male (**a**). The experimental apparatus (**b**)

The robotic fly was designed by using a 3D Computer-Aided Design (CAD) software (SolidWorks, Dassault Systemes, Vélizy Villacoublay, France) and fabricated in a biocompatible resin (VisiJet® M3 Crystal, 3D Systems) by additive manufacturing. To reproduce as much as possible the colours of *C. capitata*, nontoxic pigments were used to paint the robotic fly (Fig. 1a). A preliminary experiment to identify biomimetic traits improving the robotic fly effectiveness during interaction with *C. capitata* is reported in the Online Resource document. Iron filings (medium particle size approximately 0.420 mm) were glued in a small hole in the ventral part of the robotic fly thorax allowing magnetic actuation.

The robotic fly moved in the experimental workspace by magnetic coupling with an external robotic trajectory system located below the test arena. The robotic trajectory system was composed of two sliding axis (i.e. x and y axes) actuated by two stepper motors (i.e. Sanyo Denki 103-H7123-5040), and a Arduino Nano control board (Fig. 1b). Its operation area was of around 400 × 200 mm and with a path plotting precision of 0.01 mm. The trajectory was plotted and converted in G-Code (i.e. RS-274) before to be sent to the controlling board. An external computer (i.e. Dell XPS Intel® Core™ i7), connected to the control board, was used to manage the plotting and code conversion processes.

### Phase 1: insect–robot interaction

The experimental workspace consisted of an opaque cubic cage in Plexiglas (300 × 300 × 300 mm), with a transparent and removable top plane surface allowing inspection/access inside. The experimental workspace contained 5 discs (diameter 15 mm) obtained by citrus leaves (an host plant of *C. capitata*, Martínez-Ferrer et al. 2006) that were located in circle on a virtual circumference with diameter 150 mm.

**Fig. 2:**
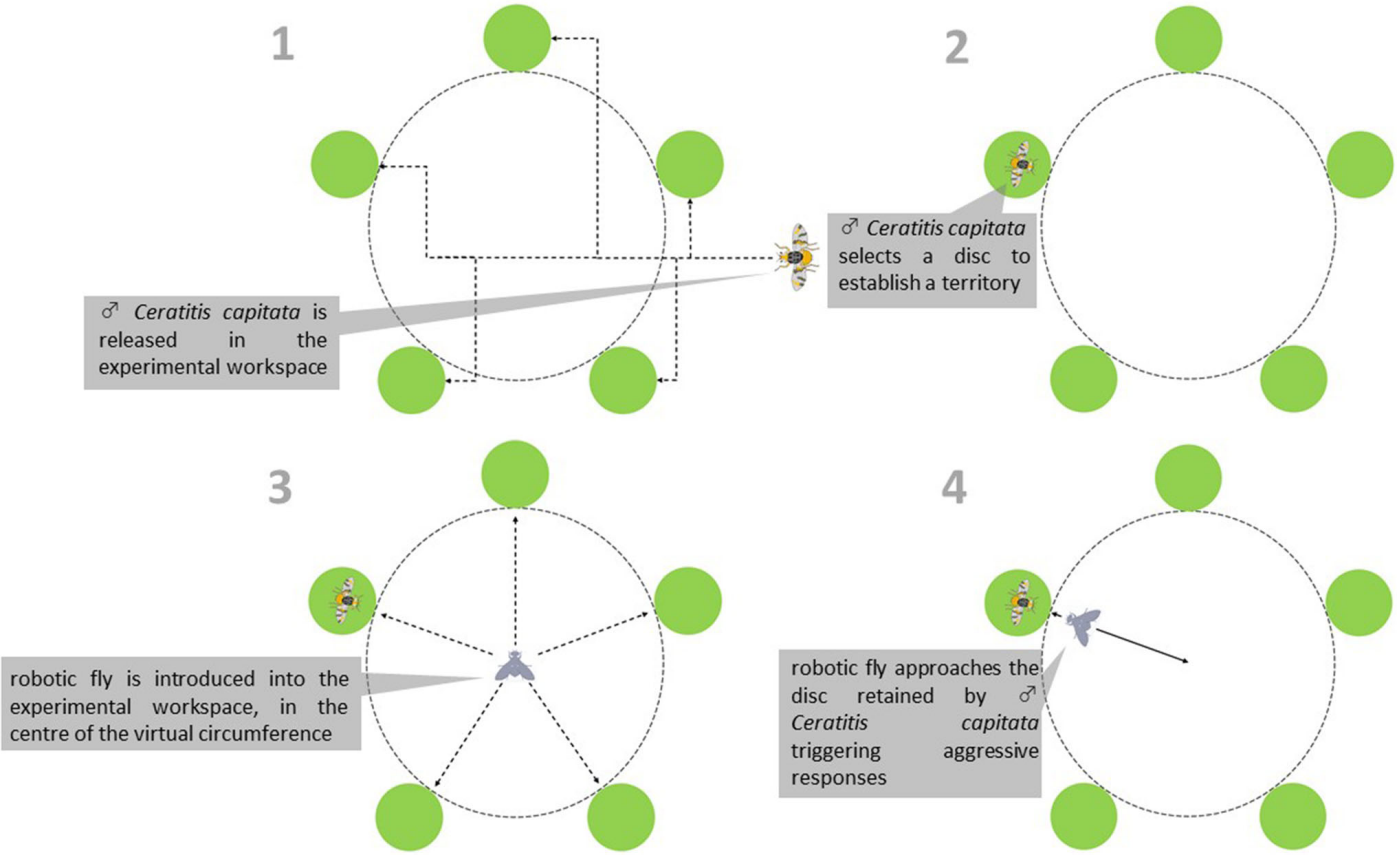
Schematic representation of the insect-robot interaction phase

Medfly adult males are quite territorial and tend to occupy a leaf or a fruit to start courting females and chasing away intruder males (Benelli et al. 2014b). So, *C. capitata* males were individually transferred into the experimental workspace and left 20 min before starting the experiment to allow the flies to establish a territory on one of the discs (Benelli et al. 2015). Then, the robotic fly was introduced into the experimental workspace, in the centre of the virtual circumference, and linearly directed towards the disc retained by a *C. capitata*. Here the robotic fly staged a conspecific intruder invading the territory of a male triggering territorial aggressive behaviours in *C. capitata* individuals (Fig. 2). Ritualized aggressive displays in *C. capitata* males include wing waving, head rocking, head pushing, wing strike, dive, boxing (see Benelli et al. 2015 for a detailed description of these aggressive traits).

The robotic fly stationed close to the disc for 30 s and then returned to its initial position for 60 s. This procedure was repeated over a period of 15 min, after which the robotic fly was removed from the experimental workspace, waiting for the subsequent experimental phase. If a fly moved to a new disc, an updated trajectory was assigned by an observer to direct the robotic fly towards the new position. Flies that not established a territory or did not engage in aggressive encounters with the robotic fly were not involved in the subsequent mating interaction phase.

### Phase 2: courtship and mating interaction

**Fig. 3:**
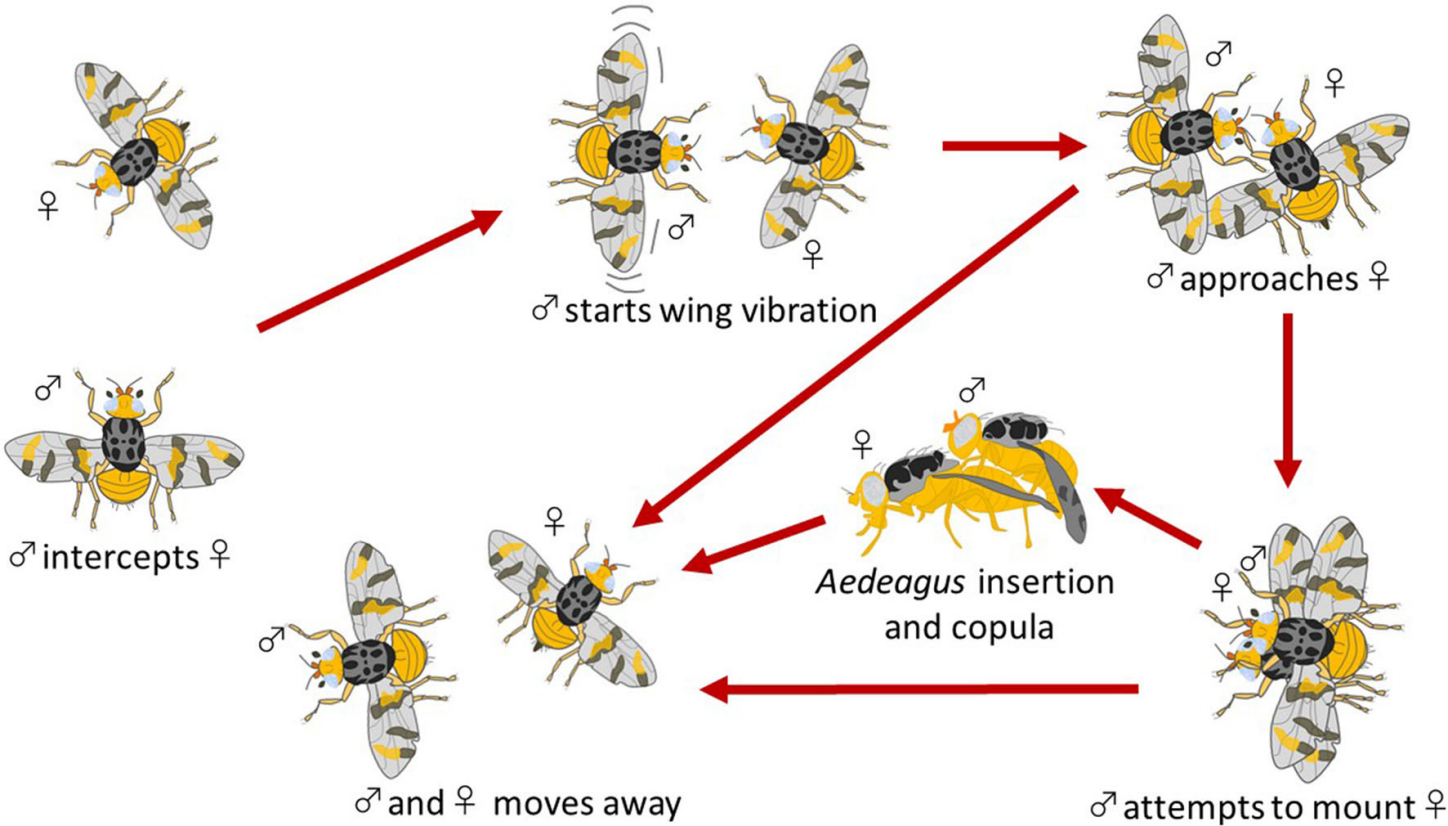
Ethogram depicting the courtship and mating behaviour of *Ceratitis capitata*

Herein, we compared the courtship performance and mating success of *C. capitata* males previously involved in animal–robot aggressive interactions, with naïve males (e.g. males not involved in previous animal–robot aggressive interactions). The observation started after a mature *C. capitata* female was released into the experimental workspace containing an experienced or a naïve conspecific male, and lasted 60 min, or until the end of the sexual interaction. The main sequences of the courtship and mating behaviour of *C. capitata* are depicted in the ethogram of Fig. 3.

For each pair of flies, we recorded the following courtship and mating behaviours exhibited by *C. capitata* (Benelli and Romano 2018): (i) the wing vibration duration, (ii) the precopula behaviour duration (i.e. from wing vibration until the male approached the female), (iii) the male mating success (i.e. copulation preceded by the successful intromission of the aedeagus), (iv) the copula duration (i.e. from the intromission of the aedeagus to genital disentanglement occurring after copulation), and (v) the whole courtship and mating sequence duration. A total of 120 experienced males and 120 naïve males were analysed.

### Statistical analyses

Data on the impact of the previous interaction with the robotic fly on the subsequent *C. capitata* males courtship and mating behaviour were neither normally distributed (Shapiro–Wilk test, *p* <  0.05) nor homoscedastic (Levene’s test, *p* < 0.05). So, we relied on nonparametric statistics to analyse data. The effects of the previous interaction with the robotic fly on the wing vibration duration, precopula behaviour duration, the copula duration, as well as the whole courtship and mating sequence duration were analysed using the Wilcoxon test (*P* = 0.05).

The impact of experience due to previous aggressive interactions with the robotic fly on males mating success was analysed by using a generalized linear model (glm) with binomial distribution: y = Xβ + ε, where y is the vector of the observations (i.e. successful or not successful mating), X is the incidence matrix, β is the vector of fixed effect (i.e. experience), and ε is the vector of the random residual effect. For the significance of differences between values, a probability level of *P* < 0.05 was used.

## Results

The effect of the robotic fly involved in previous biohybrid aggressive interactions resulted to be effective in amplifying subsequent courtship and mating behaviours in *C. capitata* males that also showed an increased mating success.

**Fig. 4:**
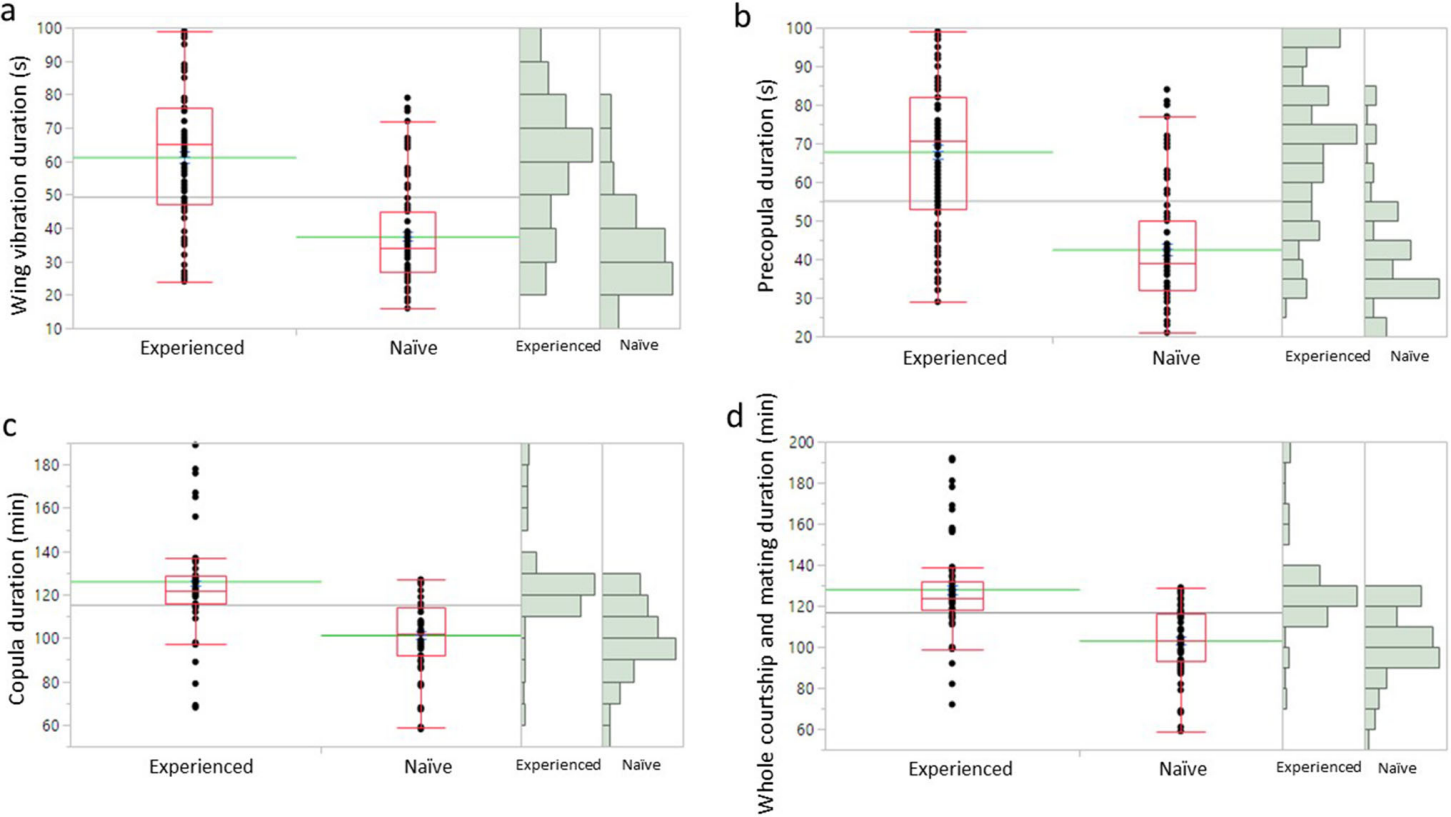
Duration of the wing vibration (**a**), pre-copula (**b**), copula (**c**), and the whole duration of the courtship and mating (**d**) in *Ceratitis capitata* males that experienced or not previous interaction with robotic flies (Wilcoxon test *P* = 0.05). In the box plots, the red lines indicate the median and their dispersion range (lower and upper quartiles, as well as outliers). The green lines are the mean, and the blue T-bars show standard error value. Data distribution is shown on histograms on the right of each box plot

Males that previously experienced aggressive interaction with the robotic fly performed the wing vibration for a significantly longer time compared to naïve males (*χ*^2^ = 74.61; *d.f.* = 1; *P* < 0*.*0001) (Fig. 4a). The duration of the precopula behaviour was significantly longer in experienced males than in naïve males (*χ*^2^ = 86.15; *d.f.* = 1; *P* < 0*.*0001) (Fig. 4b). The copula lasted more in experienced males that in naïve males (*χ*^2^ = 63.5; *d.f.* = 1; *P* < 0*.*0001) (Fig. 4c). The whole courtship and mating sequence duration was significantly longer in experienced males compared to naïve males (*χ*^2^ = 66.17; *d.f.* = 1; *P* < 0*.*0001) (Fig. 4d).

**Fig. 5:**
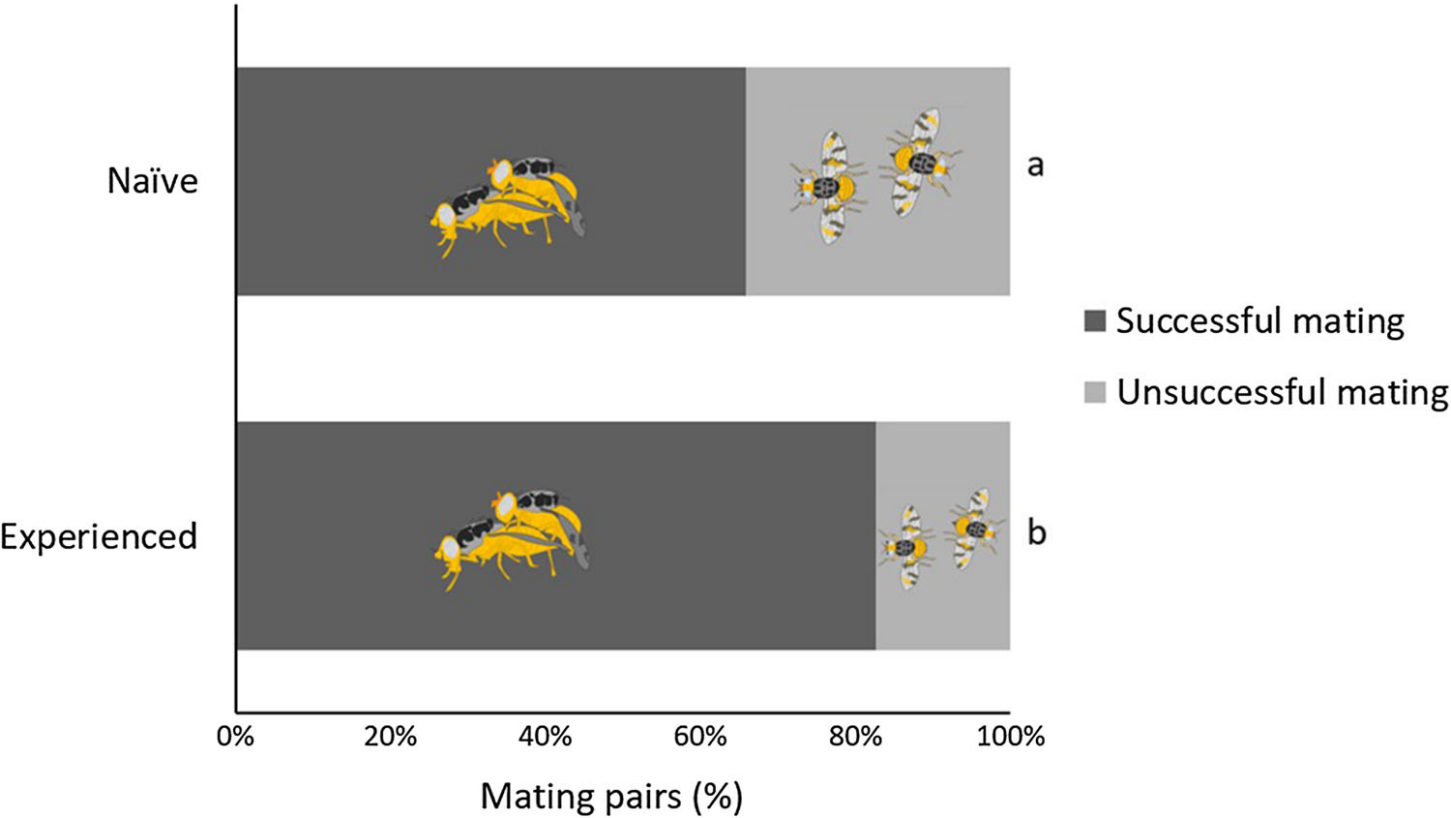
Mating success in *Ceratitis capitata* males that experienced or not previous interaction with robotic flies. *Asterisk* indicates significant differences among experienced and naïve individuals (generalized linear model, binomial distribution, *P* = 0.05)

The previous aggressive interaction experience with the robotic fly had a significant impact on male mating success (*χ*^2^ = 8.82; *d.f.* = 1; *P* = 0*.*0029), leading to a higher mating success in experienced males compared to naïve males (Fig. 5).

## Discussion

Biological control and SIT (Radcliffe et al. 2009; Sørensen et al. 2012; Garcia-Herrero et al 2018) are sustainable pest management paradigms based on domestication, handling, and mass-rearing of insect species that are then released in large numbers in the environment to predate/parasitize pest organisms (biological control), or to compete for mating with wild pest individuals (SIT). However, mass-rearing procedures reduce the fitness and performance of insects causing behavioural and physiological alterations that undermine the effectiveness and costs of such methods (Sørensen et al. 2012; Deutscher et al. 2019). In this scenario, *C. capitata* is a good model as it is a major polyphagous pest, often mass-reared for SIT purposes (Nikolouli et al. 2020), or as host for the production of endoparasitoid species exploited in biological control (e.g., Benelli et al. 2013).

Herein, robotics and bionics have been proposed as a new bioengineering paradigm to increase the reproductive performance of mass-reared strain of this insect species and thus boost its ecological performance. Some of the key advantages of using robotic replicas instead of non-focal individuals include the possibility to provide visual and physical 3D biomimetic stimuli whose chronotype coordinates can be accurately controlled (Romano et al. 2019; Bierbach et al. 2020; Worm et al. 2021; Brown et al. 2021). In addition, robotics can avoid injuries and undesired visual feedbacks to focal animals, contributing to improve reliability and standardization of experiments, as well as to ensure ethics in animal experimentation.

**Fig. 6:**
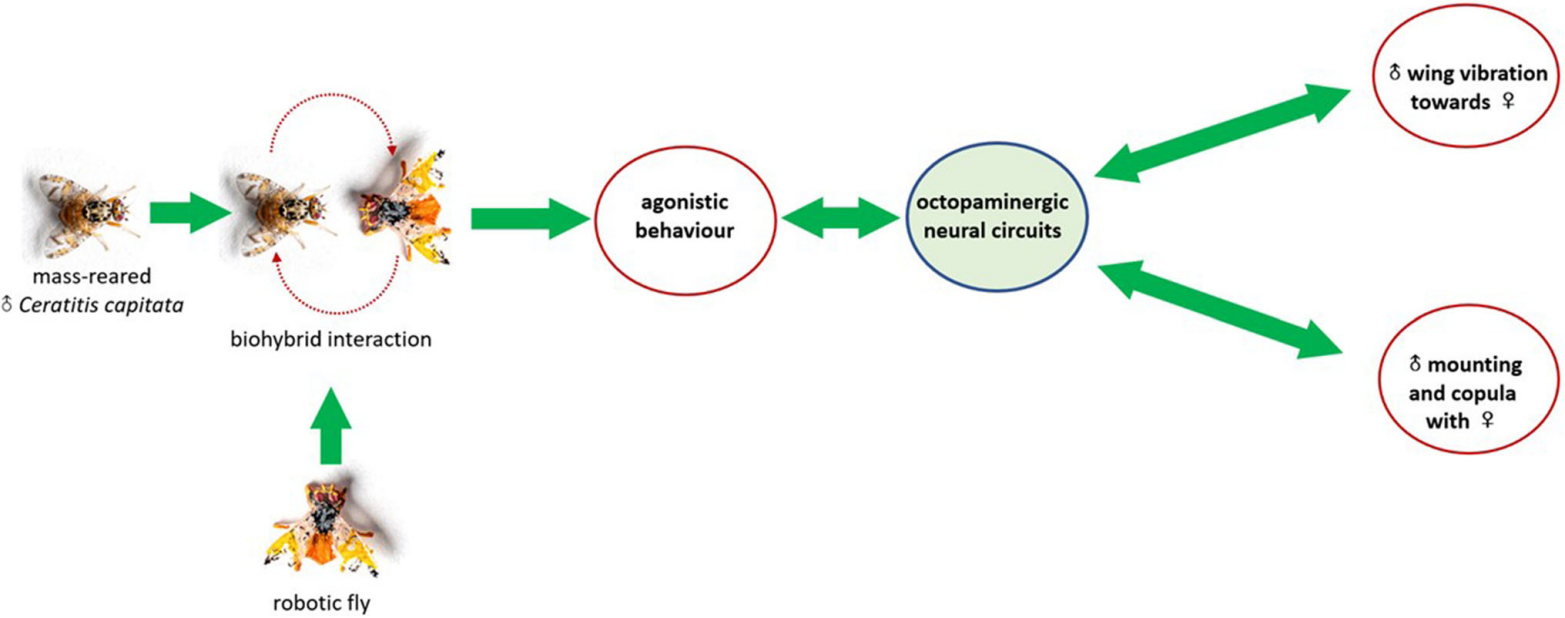
A diagram illustrating the possible feedbacks between neuronal circuits and different behaviours in *C. capitata* males post-exposure to the robotic fly

Since the territorial behaviour of *C. capitata* males is closely related to their reproductive performance (Briceño et al. 1999), the robotic fly developed here was used to interact and trigger aggressive interaction in *C. capitata* males, before mating with females, producing more competitive experienced individuals. Our findings reported a notable effect of aggressive interactions, occurring during territorial behaviour against the robotic fly, on the subsequent courtship and mating sequences. *C. capitata* males that fought with the robotic fly performed courtship displays for a longer period compared to naïve individuals. Furthermore, males involved in biohybrid aggressive encounters with the robotic fly also resulted to have a higher mating success with females. From a neuroendocrine point of view, these improved reproductive performances could be neuromodulated by octopamine (the invertebrate analogue amine of noradrenaline, acting as a neurohormone, neuromodulator, and neurotransmitter) that is surged in the haemolymph by specific octopaminergic neurones during physical exertion and fight (Adamo et al. 1995; Stevenson and Schildberger 2013). In particular, octopamine seems to be released from neurohaemal organs and the corpora cardiaca (Woodring et al. 1989; Spörhase-Eichmann et al. 1992) and can raise lipids and sugars levels in the insect haemolymph. It has been suggested that its release observed in conjunction with physical exertion may occur to mobilize energy reserves. Octopamine has also been reported to support vigorous activities in insects (Corbet 1991), whose release is directly related to the magnitude of the stimulus. Adamo et al. (1995) observed that the release of octopamine may prepare insects for prolonged activities, or to improve their recovering process after energy demanding actions. Interestingly, conspecific physical palpations, such as antennal contact, also promote the release of octopamine, appearing to be a crucial sensory cue in this mechanism. Octopamine seems to promote both aggressive and courtship motivation and learning in insects (Dyakonova and Krushinsky 2008; Stevenson and Schildberger 2013). It has been reported that, for associative learning, the sucrose reward can be substituted by the activity of single octopaminergic neurones in honey bees (Hammer 1993). In addition, different subsets of these neurons have been found to be functionally involved in the expression of aggression and courtship in Drosophilidae (Zhou et al. 2008; Certel et al. 2010; Stevenson and Schildberger 2013). So, the improved reproductive performances of the experienced *C. capitata* males may be due to the “robotic-induced” activation of dedicated sets of octopaminergic neurones during biohybrid aggressive interaction. The robotic fly, perceived as a conspecific intruder, may alter the decision-making of males that start displaying territorial traits. The presence of the robotic fly (e.g. evoking agonistic behaviours in *C. capitata* males that imply physical exertion) would act as a positive feedback for neurons expressing biogenic amines. Such condition can have beneficial effects in courtship motivation during following interaction with mature females (Fig. 6), as observed in other insects (Dyakonova and Krushinsky 2008). This study sheds light on how previous agonistic interactions with conspecifics may enhance following reproductive performances, and this may result from the increase in octopamine levels in the insect haemolymph. Further research will focus on the physiological bases of these behaviours by identifying and measuring the levels of octopamine and other neurohormones (e.g. through high performance liquid chromatography—HPLC) released during these interactions.

Recently, relevant works have reported robotics as a promising approach to modulate the behavioural response of several invasive/pest animal species, triggering in these organisms cost–benefit decision processes (Folkertsma et al. 2017; Polverino et al. 2019; Romano et al. 2017b, 2020). This study strongly contributes to the current state of the art of both IPM and bioengineering, suggesting behavioural mechanisms that can optimize insects mass-rearing procedures, as well as paving the way to the inclusion of robotics and bionics among sustainable biotechnological control techniques. To achieve real-world applications, further research should focus on the development of the robotic apparatus presented in this research in a battery configuration of smaller arenas. These multi-layer battery arenas, also including high level of automatic control, would serve as equipment in bio-farm contexts to mass-produce competitive insect males that would be simultaneously exposed in large number to biomimetic stimuli. This approach could optimize time and space use, as well as can ensure scalability of the system to fit with industrial production requirements.

## Conclusions

This research aims at establishing robotics and bioengineering as allied for the development of innovative sustainable pest management strategies via the emergent animal–robot interaction and ethorobotics paradigms.

We showed how hybrid aggressive interactions with a conspecific-mimicking robotic fly altered the behaviour of mass-reared males of the Mediterranean fruit fly *C. capitata*, boosting subsequent courtship and mating performances. Indeed, males that experienced animal–robot interactions later performed courtship displays for a longer period compared to naïve individuals, as well as had a higher mating success. Specific neuromodulators with a proven involvement in insects motivation and learning abilities may have been activated by the biohybrid aggressive interaction established in this study. In particular, the agonistic displays against the robotic fly would trigger neurons expressing biogenic amines with beneficial effects during following mating interactions with mature females. The proposed technology could be exploited to optimize insects mass-rearing procedures by modulating behavioural mechanisms.

Overall, our research shows that biomimetic robotics and ethorobotics may have a crucial role in future environmental and agricultural management approaches aimed at increasing sustainability.

## Supplementary Information

Below is the link to the electronic supplementary material.

Supplementary file1 (DOCX 200 KB)
