# Supplementary material for: How aggressive interactions with biomimetic agents optimize reproductive performances in mass-reared males of the Mediterranean fruit fly
Source: Biol Cybern. 2023 May 31;117(3):249–58. doi: 10.1007/s00422-023-00965-w (PMC10258181; doi:10.1007/s00422-023-00965-w)
Supplement: Supplementary file 1 — Supplementary file1 (DOCX 200 KB) [file 422_2023_965_MOESM1_ESM.docx]

**How aggressive interactions with biomimetic agents optimize reproductive performances in mass-reared males of the** **Mediterranean fruit fly**

**Biological Cybernetics**

Donato Romano ^a,b,^*, Giovanni Benelli ^c^, Cesare Stefanini ^a,b^

^a^ The BioRobotics Institute, Sant’Anna School of Advanced Studies, viale Rinaldo Piaggio 34, 56025 Pontedera, Pisa, Italy

^b^ Department of Excellence in Robotics and AI, Sant’Anna School of Advanced Studies, 56127 Pisa, Italy

^c^ Department of Agriculture, Food and Environment, University of Pisa, via del Borghetto 80, 56124, Pisa, Italy

*Correspondence: [donato.romano@santannapisa.it](mailto:donato.romano@santannapisa.it)

**Preliminary experiment to identify biomimetic traits improving interaction with the Mediterranean fruit fly**

To evaluate the importance of biomimetic traits for interactions with the Mediterranean fruit fly (medfly), *Ceratitis capitata,* we tested agents with different levels of biomimicry. We put to the test three agent categories: (i) the robotic fly reproducing the morphology, size, and colours of *C. capitata* adults, (ii) a white robotic fly reproducing just the morphology and size of *C. capitata* adults, and (iii) a white object of the same size as *C. capitata* adults, but with a cylindrical shape. Since medflies have been reported to frequently feed in groups in nature (Hendrichs & Hendrichs, 1990), as well as they are more attracted to food/oviposition sources where other conspecifics are already present (Prokopy et al. 2020), we tested the preference of *C. capitata* for food sources frequented by different fly-mimicking agents. The experiment consisted in a two-choice test where a food container (e.g., 60 mm diameter Petri dish containing the diet used for feeding adult medflies) was surrounded by 5 motionless agents of a category, while the other food container was without the presence of any agent. As control, we tested food containers surrounded by 5 adult medflies, each caged in a small transparent cylinder to avoid escape. For each two-choice test 50 flies were individually tested. The difference in the number of medflies feeding on different food containers was analysed with a χ^2^ test with Yates’ correction (*P* < 0.05).

Results showed that medflies preferred food containers surrounded by the robotic flies, and other conspecific medflies (S Fig. 1).


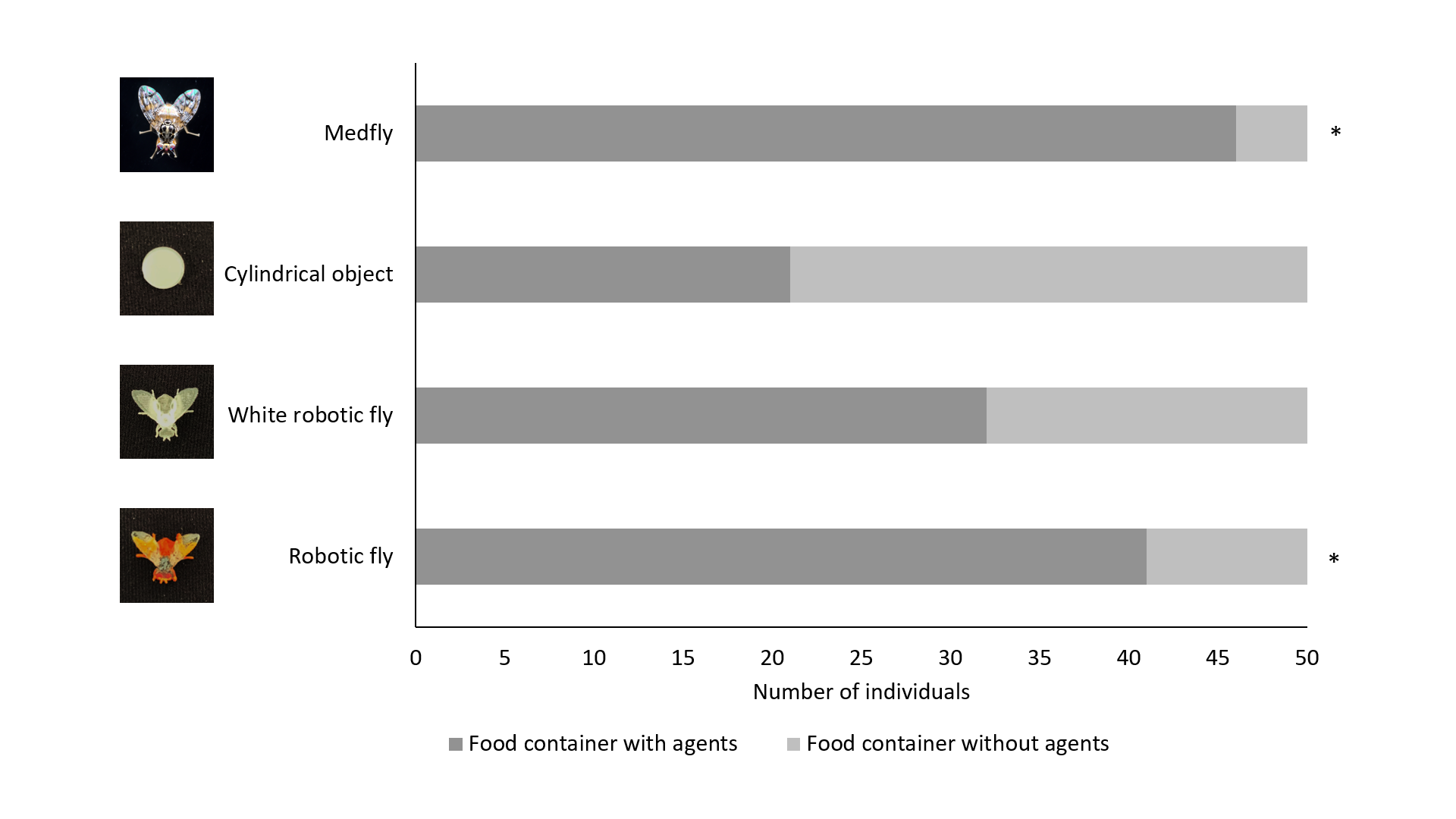


**S Fig. 1 - Number of *Ceratitis capitata* flies selecting different food containers during two-choice tests. Within each horizontal bar, the *asterisk* indicates significant differences (χ^2^ test with Yates’ correction, *P* = 0.05).**

The number of *C. capitata* selecting the food container surrounded by the robotic flies was significantly higher than the number of *C. capitata* selecting the food container without agents (41 vs. 9; χ^2^_1_= 19.22; *P* < 0*.*0001). The number of medflies preferring the food container surrounded by the white robotic flies did not differ from the number of *C. capitata* selecting the food container without agents (32 vs.18; χ^2^_1_= 3.38; *P* = 0.066). The number of medflies choosing the food container surrounded by the cylindrical object did not differ from the number of flies selecting the food container without agents (21 vs. 29; χ^2^_1_= 1.62; *P* = 0.203). The number of *C. capitata* selecting the food container surrounded by medfly conspecifics was higher than the number of medflies choosing the food container without agents (46 vs. 4; χ^2^_1_= 33.62; P < 0.0001).

Overall, these results highlight the importance of selecting the artificial agent with the greatest degree of biomimicry (e.g., the robotic fly reproducing the morphology, size, and colours of the biological model) in the core experiments of this study.

**References**

Hendrichs, J., & Hendrichs, M. A. (1990). Mediterranean fruit fly (Diptera: Tephritidae) in nature: location and diel pattern of feeding and other activities on fruiting and nonfruiting hosts and nonhosts. Annals of the Entomological Society of America, 83(3), 632-641.

Prokopy, R. J., Miller, N. W., Duan, J. J., & Vargas, R. I. (2000). Local enhancement of arrivals of *Ceratitis capitata* females on fruit mimics. Entomologia Experimentalis et Applicata, 97(2), 211-217.
